# The Concept of Ideal Lips of Caucasian Female: An Anthropometric Analysis of the Lower Third of the Face

**DOI:** 10.1007/s00266-024-04299-1

**Published:** 2024-08-28

**Authors:** Natalia Winiarska, Bartłomiej Roszkowski, Wiktor Paskal, Marcin Majak, Piotr Pietruski

**Affiliations:** 1 Medical University of Warsaw, Warsaw, Poland; 2 Center for Preclinical Research, Department of Methodology, Medical University of Warsaw, Warsaw, Poland; 3 Department of Systems and Computer Networks, Faculty of Electronics, Wroclaw University of Science and Technology, Wroclaw, Poland; 4 Department of Plastic Surgery, Center of Oncology of the Lublin Region, St. Jana z Dukli, Jaczewskiego 7 Street, 20-090 Lublin, Lubelskie Poland; 5 Ambroziak Clinic, Warsaw, Poland

**Keywords:** Lip, Face, Esthetics

## Abstract

**Background:**

The demand for lip-redefining procedures has been rising in recent years, thus creating the need for reliable and detailed reference sources on aesthetic female lips. This study investigates the morphology of the lower third of the face, including the lips and jawline, of attractive young Caucasian females.

**Methods:**

A semi-automatic photogrammetric analysis of the faces of professional female photograph models (*n*=400) of the Caucasian race aged 18–39 was performed. Angular, linear, and surface area parameters were evaluated. A graphical summarization of the average facial shape of all analyzed attractive females was generated as an average body contours (ABC) image.

**Results:**

The height of the lower third of the face equaled 0.32 ± 0.02 of the total facial height. The average lip width was 48.06 ± 3.34 mm. The upper vermilion height was found to be significantly lower than the lower vermilion height (6.47 ± 1.36 vs. 11.64 ± 1.46 mm, *p *< 0.01). The lip obliquity angle was found to be 1.05° ± 0.79°. The area surface of the upper lip vermilion was considerably smaller than the vermilion of the lower lip (*p *< 0.01).

**Conclusions:**

This is one of the largest studies on attractive Caucasian females’ lips and lower face morphology. The data it provides, including the graphical presentation of the aesthetic lower face as an ABC image, may provide physicians with valuable guidelines for lips rejuvenation and reconstruction procedures.

**Level of Evidence III:**

This journal requires that authors assign a level of evidence to each article. For a full description of these Evidence-Based Medicine ratings, please refer to the Table of Contents or the online Instructions to Authors www.springer.com/00266.

## Introduction

Lip enhancement and rejuvenation, such as volume augmentation with lipofilling and dermal fillers, as well as lip lift, are among the most commonly performed aesthetic surgery procedures in females [1–4]. The constant growth of their popularity stems from the fact that lips and perioral region play an essential role in the perception of face aesthetics. Therefore, it is crucial for aesthetic surgeons to be familiar with the factors determining the attractiveness of the perioral region.

The concept of human beauty is a very subjective matter. That is why the struggle to establish clear, objective characteristics of an attractive face has lasted since ancient times [5–7]. This attempt is hindered not only by the vague definition of beauty but also by other factors related to the cultural, gender, age, and socioeconomic background. Another potentially essential element, which seems to be rarely taken into account, is the influence of passing time on the perception of attractiveness [8]. Beauty canons were subjected to changes throughout history, which can be observed in art masterpieces of painters and sculptors of various centuries [6, 7].

Numerous studies have been conducted in search of the definition of aesthetic perioral region features. They utilized both subjective assessment methods, such as visual scales and questionaries, and objective analytic tools, including direct anthropometry, two-dimensional (2D) photogrammetry, and three-dimensional (3D) model evaluation [8–18]. Despite those attempts, there is currently no commonly accepted, objectively described concept of an attractive female lip, which creates a significant clinical issue for aesthetic and reconstructive surgery [9, 10, 19]. Little is known for example about the modern morphometric determinants of attractive lips of Caucasian (White) women. Moreover, previous studies on White females’ lips have several limitations, such as a low number of evaluated women or somewhat simple, incomplete anthropometric analysis neglecting the shape of the lips and its relation to other components of the lower face, such as jaw outline or nasal base. Finally, some analyses have been conducted many decades ago, which questions the reliability of their results today due to the cultural shift [7, 9, 11–16, 19–25].

The purpose of our study was to analyze the morphometric features of the perioral region of young, attractive White females commonly and its relations to the components of the lower third of the face. In addition, the study aimed to create a graphical representation of the attractive lip contours of evaluated females.

## Materials and Methods

### Study Material

This study was conducted in accordance with the Declaration of Helsinki and the approval of our institutional review board. A search of digital photographic website databases of ten prominent model management agencies from Europe, Canada, and the USA, Canada, was conducted. According to the criteria presented in Table 1, face photographs of professional female models of the White race were included in the study. Due to the fact that not every searched database provided information on the model’s age, after the initial selection, a photograph of each female with unknown age was assessed independently by two evaluators. The photograph was included in the study’s material only if both assessors agreed that the individual’s age was within the established age range.

**Table 1:** Inclusion and exclusion criteria for the study group

Inclusion criteria	Exclusion criteria
Female of Caucasian race	Wearing eyeglasses
The age group of 18–39 years old	Hairstyle preventing identification of Trichion landmark
Color and black-and-white photography of the face in frontal view	Makeup or outfit preventing clear distinction of facial contours and landmarks.
Relaxed, neutral expression of the face	
Open eyes and lips closed	
Head in an upright position in Frankfort’s plane	

### A.I.D. System and Assessment Protocol

Anthropometric evaluation of the models’ faces was performed with the A.I.D. system (Warsaw, Poland), a free-to-use software explicitly designed for highly standardized, complex facial and breast morphometry analysis [8, 25–27].

All gathered digital photographs were imported into the A.I.D. system. Next, using the software, the two evaluators conducted an independent semi-automatic anthropometric analysis of female faces in two sessions, with 1-week intervals and a randomized sequence of evaluated images to minimize the risk of potential memorization bias.

**Fig. 1:**
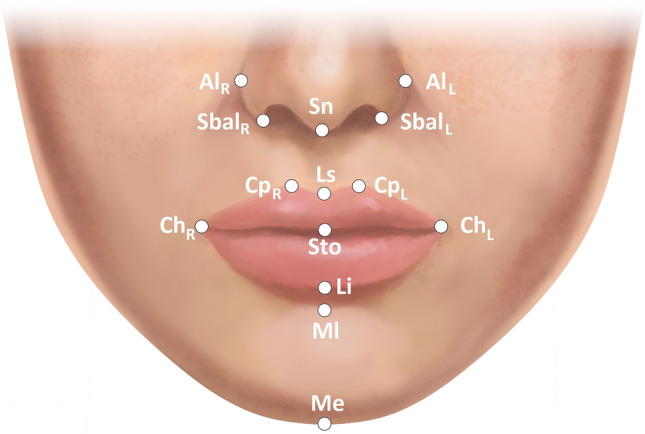
(A) Anthropometric landmarks of the lower third of the face and nose region used in the study. Descriptions of the abbreviations are presented in Table 2

**Fig. 2:**
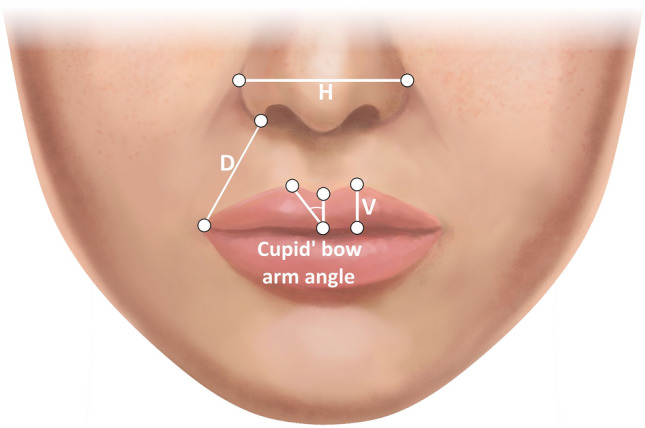
Types of investigated linear distances: (H) the horizontal distance between two landmarks; (V) the vertical distance between two points; and (D) the direct distance between two landmarks

**Fig. 3:**
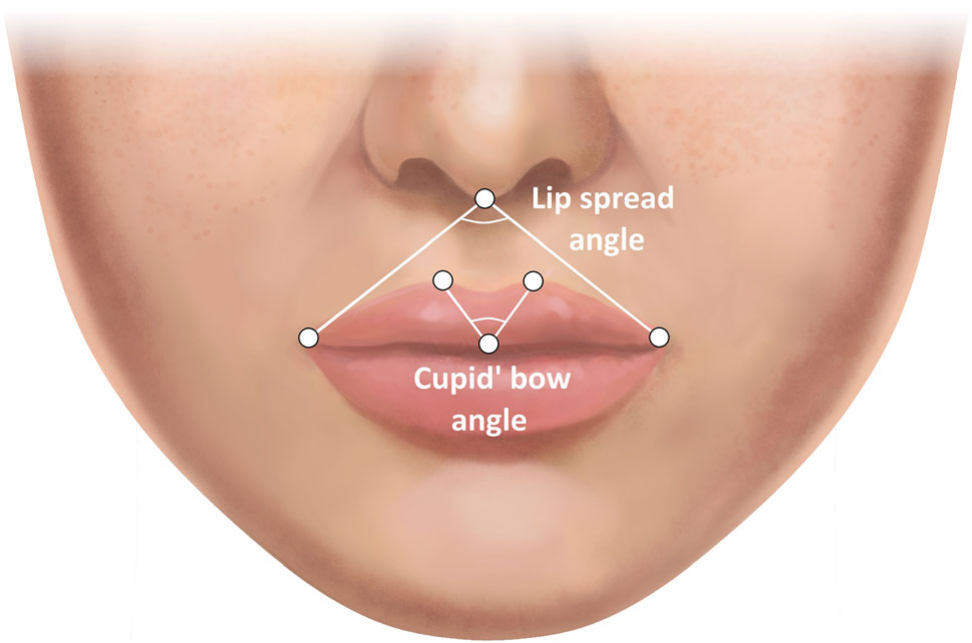
Examples of the investigated angular parameters of the lips

The photogrammetric assessment protocol utilized step-by-step labeling of specific facial landmarks and surface areas under x3 magnification view, following the instructions provided by the computer system, as described in the previous study (Fig. 1 and Table 2) [8]. For the purpose of image metric calibration (conversion of pixels to millimeters), an iris diameter was set equal to 12 mm, which is considered the mean diameter of the female iris [8, 25, 28, 29]. After the evaluator completed the landmarks identification, the A.I.D. system automatically calculated a set of angular, linear, and surface area measurements which results were exported to an Excel spreadsheet (Microsoft Corp., Redmond, WA) (Figs. 2 and 3). In addition to this numeric data, the software also generated the ABC image summarizing the contours of all evaluated female models.

**Table 2:** Summary of anthropometric landmarks used in the lower third of the face analysis

Abbreviation	Landmark	Description
ChR	Cheilion right	The most lateral point of the labial commissure on the right side
ChL	Cheilion left	The most lateral point of the labial commissure on the left side
Sto	Stomion	The midline point at the junction of the upper and lower lip vermillion
Ls	Labiale superius	The lowest vermilion point of the cupid’s bow
CpR	Crista philtri right	A point at the right peak of cupid’s bow
CpL	Crista philtri left	A point at the left peak of cupid’s bow
Li	Labiale inferius	The midpoint of the mucocutaneous border of the lower lip
Ml	Mentolabial sulcus	The midpoint of the mentolabial sulcus
Me	Menton	The most inferior midline point of the soft tissue chin
Sn	Subnasale	The midline point at the junction of the columella and upper lip skin
SbalR	Subalare right	The point at the lower inner limit of the right alar base
SbalL	Subalare left	The point at the lower inner limit of the left alar base
AlR	Alare right	The most lateral point of the right nasal alae contour
AlL	Alere left	The most lateral point of the left nasal alae contour
Tr	Trichion	The midline point at the junction of the hairline and the forehead

### Statistical Analysis

Inter-class coefficients and intra-class coefficients were used to estimate the repeatability and reproducibility of the measurements. In addition, the gauge R and R procedure was used to assess the combined contribution of repeatability and reproducibility to the total variance of the obtained results. A mean and standard deviation (SD) were calculated for every photogrammetric measurement and compared to the facial morphometric data of Caucasian professional male models described in another study [8]. The symmetry of paired anthropometric parameters was analyzed using an independent Student’s *t*-test and evaluating the values of Pearson’s linear correlation coefficients (Pearson’s r).

Proportions between the nasolabial landmarks and the jawline contour were described with mean, standard deviation, median, 10th, and 90th percentile and quartiles. In addition, a receiver operating characteristic curve (ROC) was used to estimate ratio ranges defining the proportional lower third of the face.

The statistical significance threshold was set at 5% (*p *≤ 0.05). All calculations were carried out using STATISTICA v.13 (TIBCO Software Inc., Palo Alto, CA, USA).

## Results

### Anthropometric Analysis

Analysis of the anthropometric measurements showed that inter-rater reliability (inter-class correlation) was 0.863, 0.644, and 0.727 for the linear, angular, and surface area measurements, respectively. Repeatability or intra-observer reliability (intra-class correlation) for linear parameters was 0.918. For angular and surface area measurements, the intra-class correlation was found to be 0.675 and 0.770.

The results of the photogrammetric analysis of the lower third of the face are summarized in Table 3. Table 4 demonstrates proportion indices of selected anthropometric parameters of perioral and other regions of the face.

**Table 3:** Lips and perioral region measurements

Parameter	Both sides mean ± SD	Right side mean ± SD	Left side mean ± SD	*P*-value
*Linear parameters *(mm)				
Total facial height (V)	177.92 ± 10.81			n/a
Lower third of the face height (V)	59.31 ± 3.60			n/a
Facial width–lower third of facial height level (H)	116.80 ± 6.79			n/a
Facial width–labial commissure level (H)	103.83 ± 8.00			n/a
Lip width (D)	48.06 ± 3.34			n/a
Lip width (H)	48.06 ± 3.34			n/a
Distance between cheilion and the crista philtri (D)		20.0 ± 1.8	19.4 ± 1.8	< 0.001
Distance between cheilion and the crista philtri (H)		18.2 ± 1.7	17.6 ± 1.7	< 0.001
Distance between cheilion and the labiale superius (H)		24.2 ± 1.8	23.8 ± 1.8	< 0.001
Total height of the upper lip (V)	18.74 ± 2.00			n/a
Cutaneous upper lip height (V)	12.27 ± 1.84			n/a
Upper vermilion height (V)	6.47 ± 1.36			n/a
Total height of the lower lip (V)	14.60 ± 1.84			n/a
Cutaneous lower lip height (V)	2.97 ± 1.70			n/a
Lower vermilion height (V)	11.64 ± 1.46			n/a
Chin height (V)	23.48 ± 3.14			n/a
Cupid’s bow (philtrum) width (D)	12.28 ± 2.07			n/a
Cupid’s bow (philtrum) width (H)	12.27 ± 2.07			n/a
Cupid’s bow arm width (D)		6.4 ± 1.0	6.6 ± 1.0	< 0.001
Cupid’s bow arm width (H)		6.1 ± 1.0	6.2 ± 1.0	< 0.001
Cupid’s bow arm height (V)		8.2 ± 1.3	8.3 ± 1.3	0.156
Philtrum height (V)	12.27 ± 1.81			n/a
Philtrum column height (V)		10.2 ± 1.7	10.1 ± 1.7	< 0.001
Distance between the crista philtri and the subalare (D)		12.8 ± 1.8	12.5 ± 1.7	< 0.001
Distance between the crista philtri and the subalare (V)		11.9 ± 1.7	11.8 ± 1.7	0.002
*Angular parameters *(o)				
Lip obliquity	1.05 ± 0.79			n/a
Cupid’s bow angle	141.07 ± 10.67			n/a
Cupid’s bow arm angle		70.8 ± 4.8	70.3 ± 4.4	< 0.001
Lip spread angle	106.42 ± 9.51			n/a
*Surface area parameters *(cm^2^)				
Upper lip vermilion area	28.43 ± 6.3			n/a
Lower lip vermilion area	42.67 ± 7.22			n/a

SD, standard deviation; V, vertical distance; H, horizontal distance; D, direct distance.

**Table 4:** Selected ratios of the lower face parameters

Parameters ratio	Mean ± SD
Lower third of the face height (V): total facial height (V)	0.32 ± 0.02
Lip width (H): lower face width (subnasion level) (H)	0.41 ± 0.01
Lip width (H): lower face width (labial commissure level) (H)	0.46 ± 0.03
Intercanthal distance (H): lip width (H)	0.68 ± 0.03
Nasal width (H): lip width (H)	0.69 ± 0.01
Nasal base width (H): lip width (H)	0.43 ± 0.2
Cupid’s bow (Philtrum) width (H): lip width (H)	0.26 ± 0.04
Total vermilion height (V): lip width (H)	0.38 ± 0.05
Upper vermilion height (V): lip width (H)	0.13 ± 0.03
Lower vermilion height (V): lip width (H)	0.24 ± 0.03
Philtrum height (V): lip width (H)	0.26 ± 0.04
Cupid’s bow right arm width (H): cupid’s bow left arm width (H)	0.98 ± 0.10
Total height of the upper lip (V): lower third of the face height (V)	0.32 ± 0.03
Total height of the lower lip (V): lower third of the face height (V)	0.25 ± 0.02
Chin height (V): lower third of the face height (V)	0.40 ± 0.04
Total height of the upper lip (V): total height of the lower lip (V)	1.29 ± 0.15
Cutaneous upper lip height (V): total height of the upper lip (V)	0.65 ± 0.06
Upper vermilion height (V): total height of the upper lip (V)	0.35 ± 0.06
Cupid’s bow right arm height (V): cupid’s bow left arm height (V)	1.00 ± 0.05
Cupid’s bow right arm height (V): upper vermilion height (V)	1.29 ± 0.12
Cupid’s bow left arm height (V): upper vermilion height (V)	1.29 ± 0.12
Cupid’s bow right arm height (V): cupid’s bow (Philtrum) width (H)	0.69 ± 0.15
Cupid’s bow left arm height (V): cupid’s bow (Philtrum) width (H)	0.69 ± 0.16
Philtrum height (V): total height of the upper lip (V)	0.65 ± 0.06
Philtrum right column height (V): philtrum left column height (V)	1.01 ± 0.04
Cutaneous lower lip height (V): total height of the lower lip (V)	0.20 ±0.07
Lower vermilion height (V): total height of the lower lip (V)	0.44 ± 0.08
Cutaneous lower lip height (V): chin height 0.35(V)	0.13 ± 0.05
Cutaneous upper lip height (V): cutaneous lower lip height (V)	4.15 ± 1.14
Upper vermilion height (V): lower vermilion height (V)	0.56 ± 0.10
Upper lip vermilion area: lower lip vermilion area	0.67 ± 0.13

SD, standard deviation; V, vertical distance; H, horizontal distance.

**Fig. 4:**
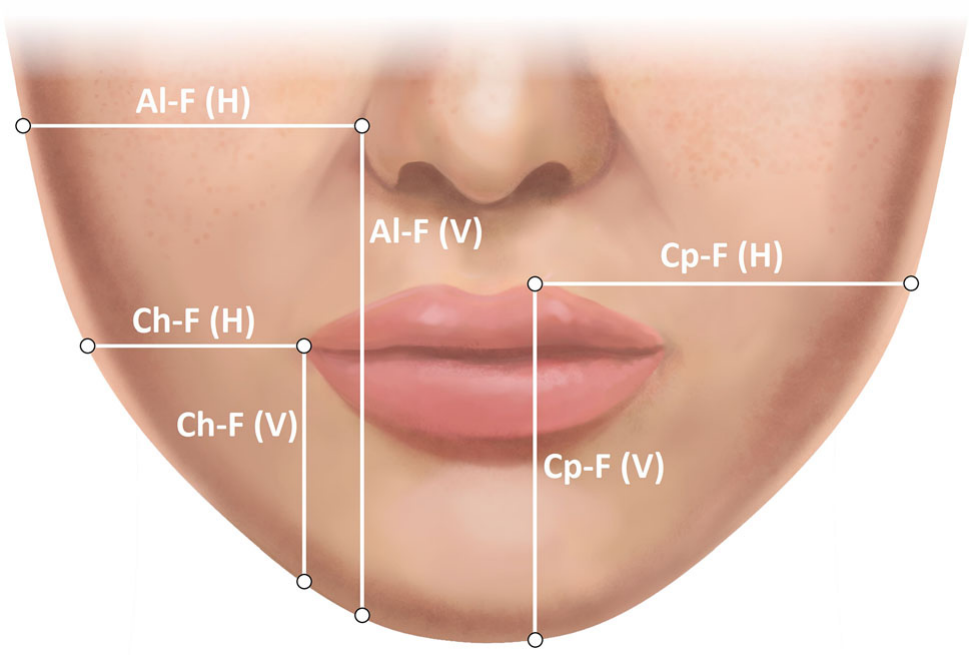
The ideal proportion of distances between the jawline contour and landmarks of the lips and nose in an attractive male face

Statistical analysis results of the proportions between the jawline outline and distinctive landmarks of the lower face are presented in Table 5 (Fig. 4). An ROC data analysis established the linear parameters ratios’ ranges characterizing feminine proportional aesthetic jawline. All six evaluated ratios’ values were within the 10th and 90th percentile range in 78.5% (*n*=314) of assessed female models (Table 6).

**Table 5:** The proportions between the jawline outline and distinctive landmarks of the lower face

Parameters ratio	Mean	SD	Min	10%	Q1	Median	Q3	90%	Max
*Average parameters proportions-all females *(*N = *400)									
Al-FR (V): Al-FR (H)	1.45	0.10	1.17	1.31	1.37	1.44	1.52	1.58	1.78
Al-FL (V): Al-FL (H)	1.45	0.11	1.19	1.32	1.38	1.45	1.53	1.59	1.87
Al-F (V): Al-F (H)	1.45	0.11	1.18	1.32	1.38	1.45	1.53	1.59	1.83
Ch-FR (V): Ch-FR (H)	1.11	0.09	0.81	0.99	1.05	1.11	1.17	1.23	1.43
Ch-FL (V): Ch-FL (H)	1.11	0.10	0.89	0.99	1.04	1.11	1.17	1.24	1.52
Ch-F (V): Ch-F (H)	1.11	0.10	0.85	0.99	1.05	1.11	1.17	1.24	1.48
Cp-FR (V): Cp-FR (H)	0.95	0.07	0.76	0.86	0.90	0.95	1.00	1.04	1.19
Cp-FL (V): Cp-FL (H)	0.96	0.08	0.76	0.86	0.91	0.95	1.00	1.06	1.24
Cp-F (V): Cp-F (H)	0.96	0.08	0.76	0.86	0.91	0.95	1.00	1.05	1.22
*Average parameters proportions-females with all ratios values in the *10* and *90th* percentile range (n=*175*)*									
Al-F (V): Al-F (H)	1.45	0.05	1.32	1.38	1.40	1.44	1.49	1.52	1.57
Ch-F (V): Ch-F (H)	1.11	0.05	1.00	1.05	1.08	1.11	1.14	1.17	1.22
Cp-F (V): Cp-F (H)	0.95	0.03	0.88	0.91	0.93	0.95	0.97	0.99	1.03
*Average parameters proportions–females without all ratios values between 10th and 90th percentile (n=*225*)*									
Al-F (V): Al-F (H)	1.45	0.10	1.19	1.32	1.37	1.43	1.53	1.58	1.76
Ch-F (V): Ch-F (H)	1.11	0.10	0.88	0.99	1.04	1.10	1.20	1.23	1.36
Cp-F (V): Cp-F (H)	0.95	0.07	0.81	0.85	0.89	0.95	1.01	1.04	1.16

SD, standard deviation; (H), horizontal distance; (V), vertical distance; Min, minimum; Max, maximum; Q, quartile; Al, alare; Ch, cheilion; Cph, crista philtri; F, lower face outline; L, left side; R, right side.

**Table 6:** The ideal proportion ranges between the male jawline contours and lips and nose landmarks

Parameters ratio	Ideal proportion range	OR (95% cl)
Al-F (V): Al-F (H)	1.37–1.54	5.58 (2.78–11.2)
Ch-F (V): Ch-F (H)	1.04–1.20	6.51 (2.75–15.4)
Cp-F (V): Cp-F (H)	0.90–1.02	21.1 (5.09–87.4)

OR, odds ratio; Al, alare; Ch, cheilion; Cph, crista philtri; F, lower face outline; H, horizontal distance; V, vertical distance.

### Average Body Contours

**Fig. 5:**
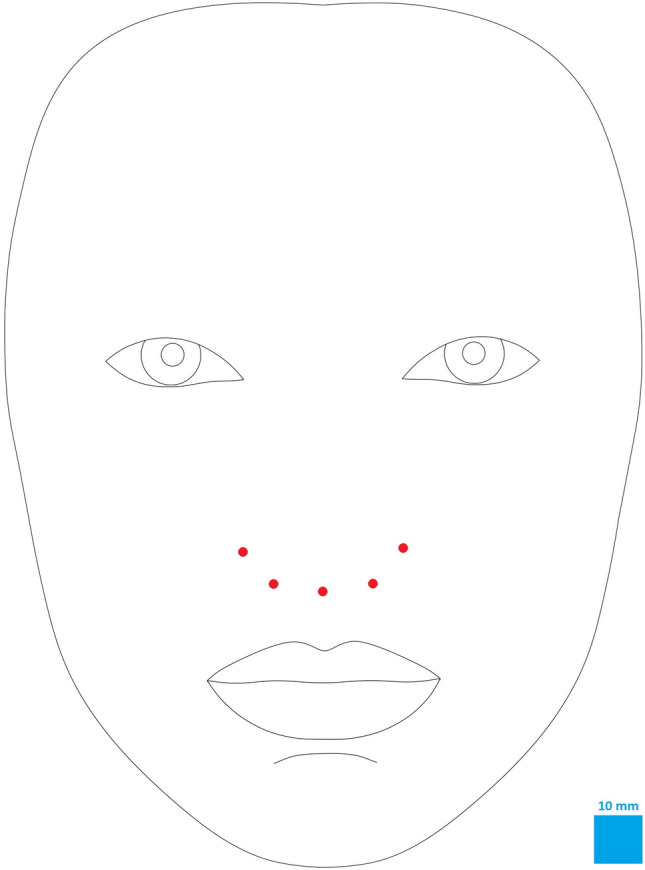
Averaged Body Contours enabling graphical summarization of all analyzed females’ faces. The lower third region components and the facial outline are presented along with palpebral fissures and nasal base landmarks

An ABC image was generated using the A.I.D. system as an alternative method of morphometric data presentation (Fig. 5). This figure enables a graphical summarization of all evaluated female faces in the form of silhouettes and respective anthropometric landmarks.

## Discussion

Growing demand for lip-redefining and reconstructive procedures, including sex reassignment surgery, has created the need for reliable reference sources on aesthetic female lips. Most of the data on this subject is provided by visual evaluations according to various assessment scales, which are subjective in nature and thus prone to low reliability and observer bias [19]. An alternative is given by anthropometric studies of the face, providing quantitative data. However, literature analysis showed that few large-scale studies objectively characterize attractive White female lips and lower faces, while most focus on normative data of a healthy population [7, 11–24].

Since beauty is a subjective term, it is not easy in assessment and quantification. Many factors may influence its perception, such as gender, age, or cultural background [7–10, 12, 30, 31] One potential solution to this problem is referring to the attractiveness judgment conducted by the fashion and marketing industry. Nowadays, due to the commonness of the internet and worldwide social media usage, the globalization process has increased [8, 32–35]. One of the results is the unification of the universal beauty canon. Thus, by using the professional model’s photographs in our study, we reduced the limitation of group selection bias due to the subjectivity of the attractiveness perception [8, 32–37]. As the individuals analyzed in our study are found attractive in general, the morphometric data’s predictive power score can be considered very high, oscillating close to a value of 1, indicating a perfect score for the White females [8].

Based on our analysis results, we create an ideal female lips concept, whose main features are presented in Table 7. It is worth noting that we conducted a complex morphometric analysis of the face of an unprecedented number of four hundred attractive females, which makes our study the largest on this subject. To better comprehend the analysis results, we calculated the ratios of selected anthropometric parameters (Table 4). Our evaluation confirms the concept of the lower third of the face, measured from the menton to the subnasale landmarks, equaling one-third of the total facial height (0.32 ± 0.02). Interestingly the mean height of the lower face found in our study (59.31 ± 3.60 mm) is significantly higher than the values (47.71 ± 3.71 mm and 48.14 ± 2.65 mm) reported by other authors[38, 39]. This finding seems to indicate that a long lower face is one of the attributes of an attractive perception of the face in White women. A relatively long chin, constituting 0.4 ± 0.04 of the lower third of the face, was observed to be characteristic of evaluated female models, thus providing another confirmation of the concept of an attractive elongated oval lower face. Therefore, not only the increase of the chin projection but also the elongation of the lower face in the vertical direction with a genioplasty or chin implant should be considered one of the major procedures to improve facial aesthetics.

**Table 7:** Features of attractive caucasian female lips and the facial lower third based on numeric morphometric data and ABC image analysis

What makes an attractive female lips and the facial lower third?
Lower face height equals one-third of the total face height.
The jawline’s has an oval shape with a narrow slope chin contour.
A relatively long chin, constituting approximately 0.4 of the lower third of the face.
The lips width is slightly lower than half of the facial width at the labial commissure level.
The lower lip vermilion is bigger than the upper lip vermilion, with its height being approximately two times larger than the upper one.
The upper lip height constitutes one-third of the height of the lower third of the face. The upper vermilion equals one-third of the upper lip’s total height.
Cupid’s bow features:
Cupid’s bow width is one-fourth of the lips width.
Longer lower vermillion and shorter upper vermillion in their central part.
Cupid’s bow arches are higher than the central part of the upper vermilion by one-third.
Symmetric localization of the Cupid’s bow arches peaks.
Cupid’s bow angle of approximatelly 141°.

For White attractive females, the rule of one-third also seems to extend to other lips proportions. The upper lip height equals 0.32 ± 0.03 of the lower third of the face height, and the upper lip vermilion height is approximately one-third of the total upper lip height (0.35 ± 0.06). The upper lip was also found to be slightly longer than the lower lip with a ratio of 1.29 ± 0.15, which is in accordance with other similar studies [22, 24, 39, 40].

The average width of attractive female lips was 48.06 ± 3.34 mm, close to previously reported values between 4.77 and 5.16 [11, 14, 16, 21, 38]. This value constituted approximately 0.41 and 0.44 of lower face width at the subnasion and labial commissure levels, respectively. Morphometric analysis showed that the ratio between the intercanthal distance and lip width ratio was 0.68. The nasal base width was 0.43 times narrower than the lip, significantly lower than the ratio of 0.75 reported in average-appearance White women by Sawyer et al [14]. Following this, set of proportions might be useful in planning lip surgery such as commissuroplasty or reconstruction of the perioral region. We find the correlation of the lips and nose appearance on the esthetic perception of one another to be a fascinating topic for future research, as it seems that lower nasal base to lips widths ratio is a more favorable facial beauty feature.

Since vermilion plays a significant role in the perception of facial appearance, most perioral rejuvenation or reconstructive studies are focused mainly on lip component handling [8, 11, 13]. Yet, there is surprisingly little detailed information on attractive White female vermilion features, especially Cupid’s bow characteristics. Our analysis shows that upper vermilion is almost 2 times shorter than lower vermilion (6.47 ± 1.36 mm vs. 11.64 ± 1.46 mm, *p *< 0.005) and constitutes one-third of the total height of the upper lip. Anthropometric studies conducted on White women of ordinary beauty showed similar central lengths of upper (8.5-9 mm) and lower (8.4-10 mm) vermillion [14, 15, 20, 21]. The lower vermillion height, on the other hand, equals approximately 1.56 ± 0.08 of the upper lip height. The surface area proportion between the lower and upper lip vermilion equals 1.33 ± 0.13. Both findings disprove the golden ratio concept, claiming those values should equal 1.618 [10]. According to our analysis, attractive females tend to have longer lower vermillion and shorter upper vermillion in their central part, corresponding to the high Cupid’s bow arches. Therefore, procedures such as lip lift or volumetric augmentation should be aimed at preserving the proportions mentioned above between the lower and upper vermillions and between the specific Cupid’s bow components.

**Fig. 6:**
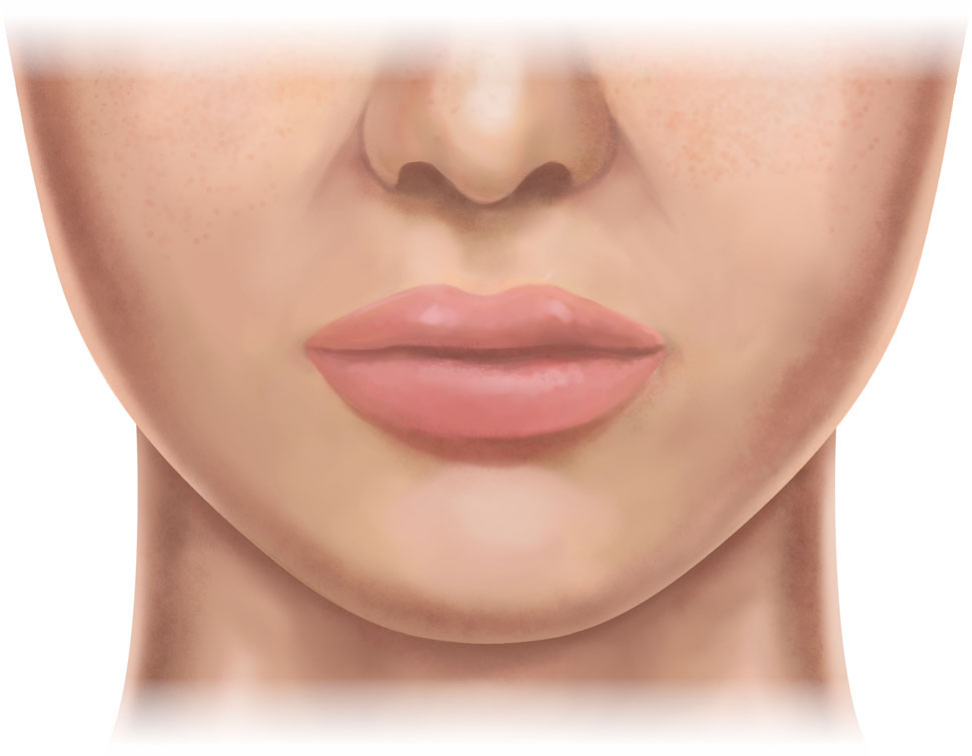
An artistic graphical presentation of the ideal female’s lower face based on a generated ABC image

The esthetic Cupid’s bow structure analysis demonstrated an average height of its arm of 8.3 ± 1.3 mm, which is higher than in males (7.95 mm, *p *< 0.05) who are considered attractive [8]. This value is also significantly higher than Cupid’s bow arms of average White females (7.2 mm) reported by Sawyer et al [14]. The desired ratio of this parameter to the upper vermilion height should equal 1.29. The observed in our study, Cupid’s bow width was 12.27 ± 2.07 mm, which should constitute approximately 0.26 ± 0.04 of lip width. The attractive Cupid’s bow angle was found to be 141.07° ± 10.67o with an individual arm angle of approximately 70° (Table 3). The detailed contours of the attractive female Cupid’s bow are presented in Figs. 5 and 6.

According to the literature, the ideal philtrum height in females should be between 12 and 15 mm [20, 42–44]. The esthetically desired ratio between the height of the philtrum and that of the vermillion upper lip, also referred to as philtral labial score (PLS), should be within the 1.2–2.3 range [20, 45]. Our analysis indicates that the ideal value of the PLS parameter is 1.9.

Interestingly, the statistical analysis showed that the value of nine out of ten paired parameters of the perioral region was significantly different (*p *< 0.005). This finding proves that the human face is not ideally symmetrical, and this should be mentioned to the patients during preoperative consultations. This also proves the claim of other studies that facial attractiveness is influenced mainly by proper proportions balance and not symmetry [5, 8, 9, 11, 13, 41]. The only evaluated parameter that demonstrated the insignificant difference in value on both sides was the height of Cupid’s bow arm (*p *= 0.156). This observation indicates that this parameter-side imbalance may negatively impact the perception of lip attractiveness. Therefore, achieving the symmetrical Cupid’s arms peaks should be one of the primary goals in lips esthetic procedures, such as lip lift or volumetric augmentation.

Based on the performed quantitative analysis, the AID system generated the ABC image, representing the averaged contours of the jawline and lips of an attractive White female. Figure 6 presents the artistic visualization of such a face. A clear pattern of elongated and oval shape of the lower third of the face can be noticed. Another characteristic feature easily observed is Cupid’s bow arms with ends at the same level.

**Fig. 7:**
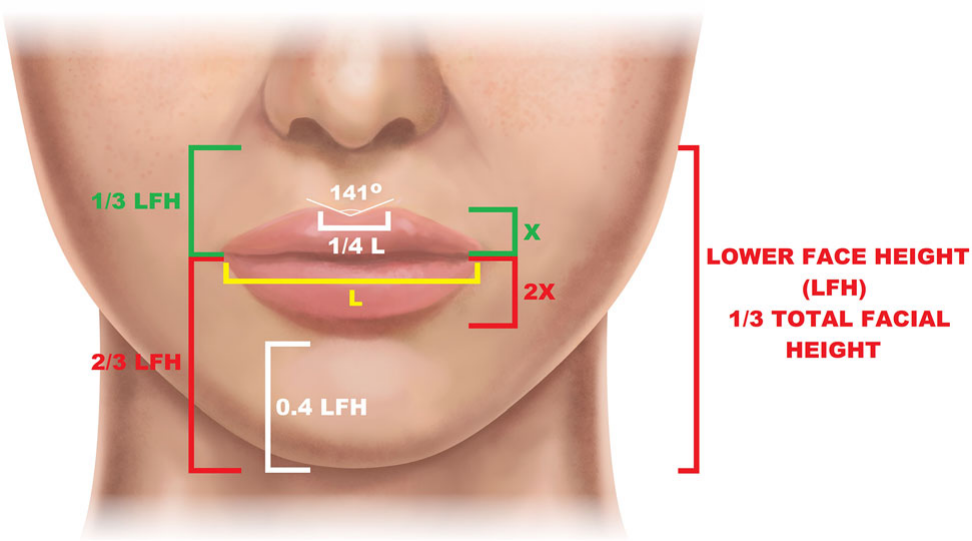
An artistic graphical presentation of the ideal female’s lower face with the most significant morphometric ratio values. (L) lips width; (LFH) lower face height; (X) Upper lip vermilion

Based on the performed quantitative analysis, the AID system generated the ABC image, representing the averaged contours of the jawline and lips of an attractive White female. Figure 5 presents the artistic visualization of such a face. A clear pattern of elongated and oval shape of the lower third of the face can be noticed. This differs from the conclusion of a recent study on attractive White males who tend to have a broad, horizontally shaped lower chin segment of width considerably similar to the lips width [8]. Another characteristic feature of the female’s lip is Cupid’s bow arms with ends at the same level. It is worth noticing that the attractive shape of the lower face is much more consistent in women than in men. Statistical analysis showed that over 89% of evaluated female faces had similar, which can also be referred to as ideal, values of proportions of lower face-defining parameters (Table 6, Fig. 7). On the other hand, the perfect male lower face contour is less unified, with only 40% of men considered attractive sharing all contour-defining parameters within the ideal range value [8].

Our study is not without limitations. One of them is that only photographs in the frontal view were evaluated. Due to the lack of standardized lateral-view images of professional models in the searched databases, we could not analyze the level of lip projection, as well as chin and submental contour lines. Some will also find as a limitation the fact that 2D photographs were assessed and not 3D models. However, it has been demonstrated that morphometric analysis of 2D images offers similar measurement precision and reliability as the one conducted on 3D models. The only unquestionable advantage of 3D models is the possibility of volumetric assessment and more precise surface area evaluation. Nevertheless, such analysis is not possible due to the lack of an open-access database containing a large number of standardized 3D images of professional models.

Another limitation is the lack of precisely defined mean age of evaluated individuals, as not all searched photographic databases provided information about the model’s age. Instead, we used the group age span of 18–39, confirmed by subjective analysis of each face of unknown age conducted by two independent observers. The photograph was included in the study only if both raters agreed that the female was in the approved age range.

One must be aware that our findings and conclusions may not be universal for non-White females since facial morphological features may vary significantly among races [12, 13, 30, 31]. Therefore, the results of our study should be primarily used as surgery planning guidelines for White females. As demonstrated [46], additional detailed studies are needed to establish the morphometric data of the perioral region of attractive females of other races. We plan to conduct such studies in the near future. It must also be noted that the beauty concept is a flexible term that evolves over time along with fashion and artistic trends [6, 7, 9, 32–37]. That is why similar studies should be conducted every few decades to have reliable insight into facial attractiveness-defining features.

## Conclusions

Our study results, obtained from professional female models analysis, demonstrate a detailed concept of attractive lips and the lower third of the face of White women. The graphical summarization of the anthropometric data as an ABC image is especially worth noting since it allows fellow researchers and clinicians to comprehend it easily. This insight into factors influencing lips esthetics may be used to support the planning of both the lips’ esthetic and reconstructive surgical procedures.
